# Tuneable Acidity in Fluorinated Al-SBA-15 Materials for the Esterification of Valeric Acid to Alkyl Valerates

**DOI:** 10.3389/fchem.2020.00042

**Published:** 2020-01-31

**Authors:** Miguel Blanco-Sánchez, Evan Pfab, Noelia Lázaro, Alina M. Balu, Rafael Luque, Antonio Pineda

**Affiliations:** ^1^Departamento de Química Orgánica, Universidad de Córdoba, Córdoba, Spain; ^2^Department of Chemistry, People's Friendship University of Russia (RUDN University), Moscow, Russia

**Keywords:** heterogeneous catalysts, mesoporous materials, biomass valorisation, esterification, alkyl valerates

## Abstract

The acidity of Al-SBA-15 materials functionalized by ball milling with several niobium loadings (0. 25–1 wt.%) as well as with several fluorine loadings (by wet impregnation using NH_4_F as a precursor) was characterized and materials investigated in the esterification of valeric acid to alkyl valerates. The parent Al-SBA-15 support as well as the modified materials loaded with Nb and/or F have been catalysts synthesized characterized by X-ray diffraction (XRD), N_2_ physisorption measurements, and diffuse reflection infrared spectroscopy (DRIFT) among others. A special interest was paid on the acidity of the materials that was investigated by temperature-programmed desorption of pyridine. Interestingly, the characterization results for the materials containing fluorine showed up an increase in the acidity strength despite of a reduction in the number of acid sites. The catalytic performance of the as-prepared catalysts was investigated in the microwave-assisted esterification reaction of valeric acid to valerate esters. Thus, while the materials modified with niobium exhibited a lower catalytic activity as compared with the catalytic support (Al-SBA-15), the materials loaded with fluorine either onto Al-SBA-15 or on Nb1%/Al-SBA-15 materials presented enhanced conversion values of valeric acid. Therefore, it can be said that the new acid sites with enhanced strength formed by the incorporation of fluorine boost the esterification of valeric acid with alcohols to form the respective valerate ester.

## Introduction

Alternative feedstocks for fuels and chemicals are critically necessary to address the environmental concerns regarding to the increase on carbon emissions and the natural resources depletion, along with a world population that is constantly growing (Lange et al., 2010; Ho et al., 2014). Biofuels and other biomass valorization routes have emerged as sustainable solutions to address such issues and have since been a major focus in the field of green chemistry (Lange et al., 2010; Ho et al., 2014; Sheldon, 2016). In this sense the sustainable design of efficient catalytic materials in the transformation of such biomass plays a key role.

Initially, first generation biofuels were introduced and developed from crops such as sugarcane, oilseeds, and starch (Naika et al., 2010). First generation biofuels were met with much skepticism amongst the scientific community due to an environmental impact that was not significantly reduced, a poor carbon footprint and a potential increase in the price of food (Naika et al., 2010; Palkovits, 2010). In addition, there were ethical issues due to the fact that the crops used for feedstocks were in direct competition with food production and could not meet biofuel demands without cutting into a large portion of the world's limited food supply. Thus, an additional problem was created in attempt to solve an existing one (Palkovits, 2010).

A new generation of biofuels was explored to address previous ethical limitations. Namely, routes that employed feedstocks without food interest and not in direct competition with the food industry were investigated, merging lignocellulosic biomass as a viable option (Palkovits, 2010). Aside from lack of food interest, the use of lignocellulosic biomass is also advantageous for its worldwide accessibility, low price, and minimal impact on the environment (Palkovits, 2010). Among other chemicals lignocellulosic biomass is an important source of levulinic acid that can be obtained via the hydrolysis of the cellulose fraction (Palkovits, 2010). Such levulinic acid via successive hydrogenation and dehydration/cyclization reactions can be transformed into valeric acid that can then undergo esterification to form alkyl valerate, a valuable product that can also be used as a biofuel, among other applications (Lange et al., 2010).

Traditionally, homogeneous catalysts from mineral inorganic acids have been used to carry out such esterification reaction, including hydrochloric acid, sulfuric acid or nitric acid, and even organic acids such as p-toluenesulfonic acid (Carmo et al., 2008; Srilatha et al., 2009). However, the use of homogenous catalysts leads to a wide range of disadvantages related with their handling as well as toxicity and corrosiveness. In addition, they are difficult to separate from the reaction medium, which complicates their reuse and leads to higher production costs (Park et al., 2010). In contrast, heterogeneous catalysts offer many environmental improvements over traditional homogenous catalysts including higher activity, easier handling, and better separation from the reaction mixture (Lange et al., 2010; Park et al., 2010; Borges and Díaz, 2012). Among heterogeneous catalysts, solid materials including sulfonated resins (Mo et al., 2008), zeolites (Doyle et al., 2016), and metal oxides (ZrO_2_, Nb_2_O_5_) (López et al., 2008; Rade et al., 2018) are well-known catalysts for the biofuels production through esterification reactions. In particular, for the esterification of valeric acid to produce valerate fuels the use of zeolites modified with cobalt and platinum (Kon et al., 2014; Sun et al., 2014), vanadium supported on silicates (Udayakumar and Pandurangan, 2014), cationic resins (Sharma et al., 2014), and supported enzymes (Corradini et al., 2016; Cebrián-García et al., 2018a,b) have been widely investigated in literature.

The use of mesoporous materials has received a special attention as they are known to be especially useful as catalytic supports. In this sense the SBA-15 type materials, which own a two-dimensional and hexagonal structure (Stuckv et al., 1998; Zhao, 1998; Zhao et al., 2014), is one of the most widely used mesoporous silicates due to their great stability, both thermal and hydrothermal, and the large number of mesoporous channels and thick mesoporous walls (Kresge et al., 1992) that makes them a suitable material in reactions where large molecules participate. However, SBA-15 silicates require heteroatoms and/or the incorporation of different functionalities onto their surface to make them catalytically active (Luque et al., 2012). Such functionalization can be carried out by different methodologies, either direct synthesis or by post-synthesis approaches, which include impregnation, incipient wetness, and other recent methodologies such as mechanochemical grinding (Pineda et al., 2011), which is an efficient procedure that minimizes the use of solvents, which has environmental benefits (Pineda et al., 2018).

Interestingly, mesoporous silicates with SBA-15 structure modified with niobium by conventional methods have been shown to be effective in different esterification reactions, such as propionic acid (Silva et al., 2017) or ricinoleic acid (García-Sancho et al., 2017). Niobium oxide (Nb_2_O_5_) is expected to form on the silica supports when it is introduced with the precursor (Jehng and Wachs, 1991), leading to the formation of Si—O—Nb bonds that exhibit Brönsted acidity (Xu et al., 2002), an important factor in the activation of both the carboxylic and the alcohol during esterification reaction (Silva et al., 2017). Similarly, the treatment of mesoporous aluminosilicates with ammonium fluoride (NH_4_F) can enhance the acid properties of the synthesized materials (Luque et al., 2005). This has been attributed to the formation of Si(O_2_F)OH species in the vicinity of silicon atoms (or aluminum in some cases) of the network. However, if treatment with ammonium fluoride is carried out under harsh conditions deterioration in the mesoporous structure could be produced (Luque et al., 2005).

In this work, SBA-15 aluminosilicate, with a theoretical Si/Al molar ratio of 20, have been modified with different loading of niobioum and/or ammonium fluoride via mechanochemical grinding and wet impregnation, respectively, for their use as catalysts in the microwave-assisted esterification of valeric acid toward the formation of valerate fuels. An important attention have been paid on the modification of the acidic properties of the investigated material after the different modifications that the material underwent.

## Materials and Methods

### Materials Synthesis

Al-SBA-15 materials used as catalytic support were synthesized with a Si/Al molar ratio of 20 using a sol-gel approach previously reported (Jarry et al., 2006). Thus, 8 g of copolymer triblock Pluronic 123 (P123) (Sigma-Aldrich, St. Louis, MI, USA) used as surfactant, were dissolved in 300 mL HCl solution (pH = 1.5) stirring for 2 h. Then, 18 mL of tetraethyl orthosilicate (Sigma Aldrich) were dropwise added, followed by the addition of the appropriate amount of aluminum isopropoxide to achieve a Si/Al molar ratio of 20. The mixture is kept under stirring for 24 h at 35°C and, subsequently, underwent hydrothermal treatment for 24 h. Finally, the sample was filtered out and calcined at 600°C for 8 h to ensure the complete removal of the template.

Firstly, Nb-modified Al-SBA-15 materials with different metal loadings (0.25, 0.5, 1 wt.%) were obtained using a mechanochemical protocol already described by our research group (Pineda et al., 2018), employing niobium ammonium oxalate hydrate as salt precursor (Sigma-Aldrich). The materials were prepared in a Retsch PM-100 planetary ball-mill using a stainless-steel container, in which the Al-SBA-15 support and the metal salt precursor were ground together at 350 r.p.m. for 10 min. The material obtained was calcined at 400°C for 4 h, with a heating rate of 5°C min^−1^ and named NbX%/Al-SBA-15, where X is the theoretical metal loading.

Finally, fluorine modified materials were synthesized through a well-known wet impregnation protocol. Thus, the materials Nb1%/Al-SBA-15 and Al-SBA-15 were impregnated with different amounts of NH_4_F to achieve fluoride loadings of 3 and 10 wt.% for each material. Eventually, the materials were calcined at 400°C for 4 h, to obtain the following materials: F3%-Nb1%/Al-SBA-15, F10%-Nb1%/Al-SBA-15, F3%/Al-SBA-15, and F10%/Al-SBA-15.

### Characterization Techniques

The textural properties of the as-synthesized materials have been obtained by the nitrogen adsorption/desorption isotherm at liquid nitrogen temperature (77 K) using a Micromeritics ASAP 2000 porosimeter (Micromeritics Instrument Corp, Norcross, GA, USA). Before analysis, samples were outgassed for 12 h at 130°C. The specific surface area was evaluated using the linear part of the Brunauer, Emmet, and Teller (BET) equation in the interval 0.05 < P_0_ > 0.22. The pore sized distribution was calculated from the adsorption branch and using the Barret, Joyner, and Halenda (BJH) equation.

The arrangement of the synthesized catalysts has been evaluated using the X-ray diffraction technique. X-ray diffractograms were acquired in a Bruker D8D Discover (40 kV, 40 mA) diffractometer (Bruker AXS, Karlsruhe, Germany) which allows particle size measurement and phase ratio determination. The radiation employed was the line Cu Kα (λ = 1.54 Å) and the goniometer speed was 0.5°/min in the interval 0.5° < 2Θ > 5° and 1°/min for the diffractograms recorded in the range 10° < 2Θ > 80°.

Diffuse reflection infrared spectroscopy (DRIFT) spectra were acquired using an ABB MB3000 spectrometer fitted with an environmental chamber (Diffus IR™ Pike Technologies). The spectra were recorded with a resolution of 8 cm^−1^ in the interval 600–4,000 cm^−1^.

The acid properties of the synthesized materials were evaluated using a pulse chromatographic titration method using pyridine and 2,6-dimethylpyridine as probe molecules, for total and Brönsted acidity, respectively, following a methodology similar to the one previously reported by our research group (Sheldon, 2016).

In addition, temperature-programmed desorption of pyridine (Py-TPD) has also been used for the characterization of the investigated material. For such measurement a modified gas chromatograph modified with an empty column placed before the reactor where the catalyst is located and then, directly connected to the flame ionization detector (FID). The carrier used was pure N_2_ with a flow rate of 50 ml min^−1^. Py-TPD experiments were performed in the 323–873 K range once the sample was completely saturated with pyridine.

The elemental composition of the materials herein investigated was measured by means of Inductively Coupled Plasma Mass Spectrometry (ICP-MS) in a Perkin Elmer NexionX fitted with a quadrupole mass detector. Prior to the analysis the samples were digested at room temperature in a mixture acids.

### Catalytic Activity

The catalytic activity of the materials was evaluated in the microwave-assisted esterification of valeric acid with different alcohols such as methanol and ethanol. In a typical reaction, 50 mg of catalyst, 2 mL of ethanol, 0.1 mL of valeric acid were employed and submitted to 300 W microwave irradiation power for 15 min, reaching pressures in the reaction vessel below 50 PSI. Finally, the reaction mixture was analyzed using an Agilent 7890GC fitted with a Petrocol DH capillary column (100 m × 0.25 nm × 0.5 μm) and an FID detector.

## Results and Discussion

**Figure 1:**
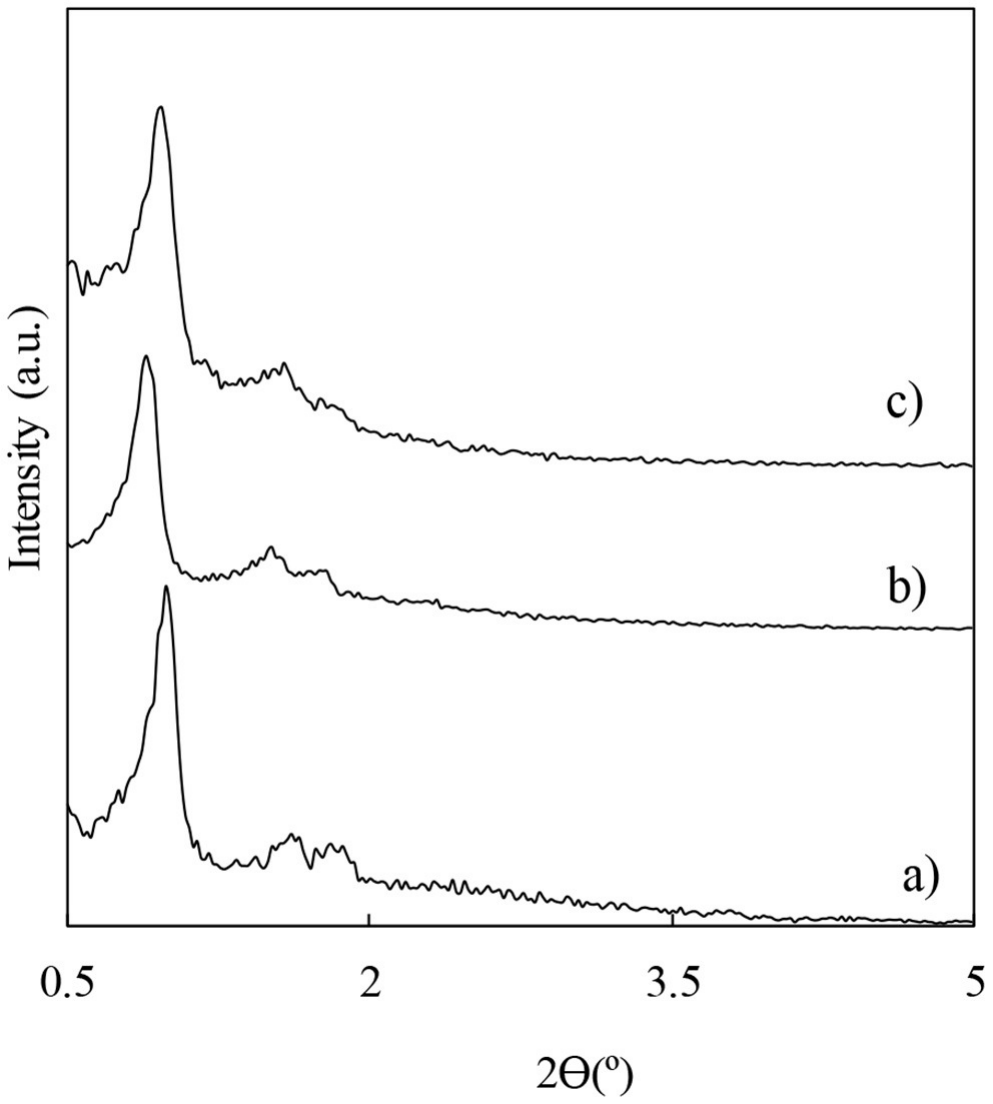
Low-angle X-ray diffractograms for the material used as catalyst support Al-SBA-15 (a), and for the materials obtained with different fluorine loadings: F3%/Al-SBA-15 (b) and F10%/Al-SBA-15.3 wt.% (c).

Several aluminosilicate materials with structure type SBA-15 were loaded with different loadings of niobium. Alternatively, Al-SBA-15 as well as selected Nb-modified aluminosilices samples were modified with different loadings of fluorine by wet impregnation using ammonium fluoride as precursor (Table S1). The aim of the modification with both niobium and fluorine is to boost the acidity of the materials in order to get materials catalytically active in the esterification of valeric acid. The incorporation of niobium on to the Al-SBA-15 material used as support was performed by ball-milling that is a method developed by our research group featuring advantages including an easy and simple preparation method for supported metal nanoparticles. Another important advantage of this methodology is the fact that the metal deposition occurs on the external support surface that facilitates the access of bulky molecules to the active sites (Pineda et al., 2011). The influence of the different modifications as compared with the parent support were investigated through different characterization techniques. Firstly, the impact on the typical hexagonal arrangement in SBA-15 materials was assessed by low-angle X-ray diffraction (Figure 1). In all the cases, the typical diffraction lines expected for SBA-15 materials, one sharp reflection (100) and other two weaker corresponding to the planes (110) and (200), are preserved even for those materials with a higher fluorine loading such pattern is still distinguishable. The treatment with ammonium fluoride leads to a decrease in the diffraction peaks, which is more remarkable for those materials modified with a 10 wt.% of fluorine, preserving the hexagonal arrangement due for this material type. In addition, X-ray diagrams for the niobium loaded materials were also acquired (Figure S1) and the materials also shown the distinctive lines expected for SBA-15 type materials as a good indicative of the materials stability toward the treatment that underwent. In addition, the identification of the Nb phase present in the as-synthesized materials was not possible due to the low loading of this metal in the materials herein investigated (Figure S3).

**Figure 2:**
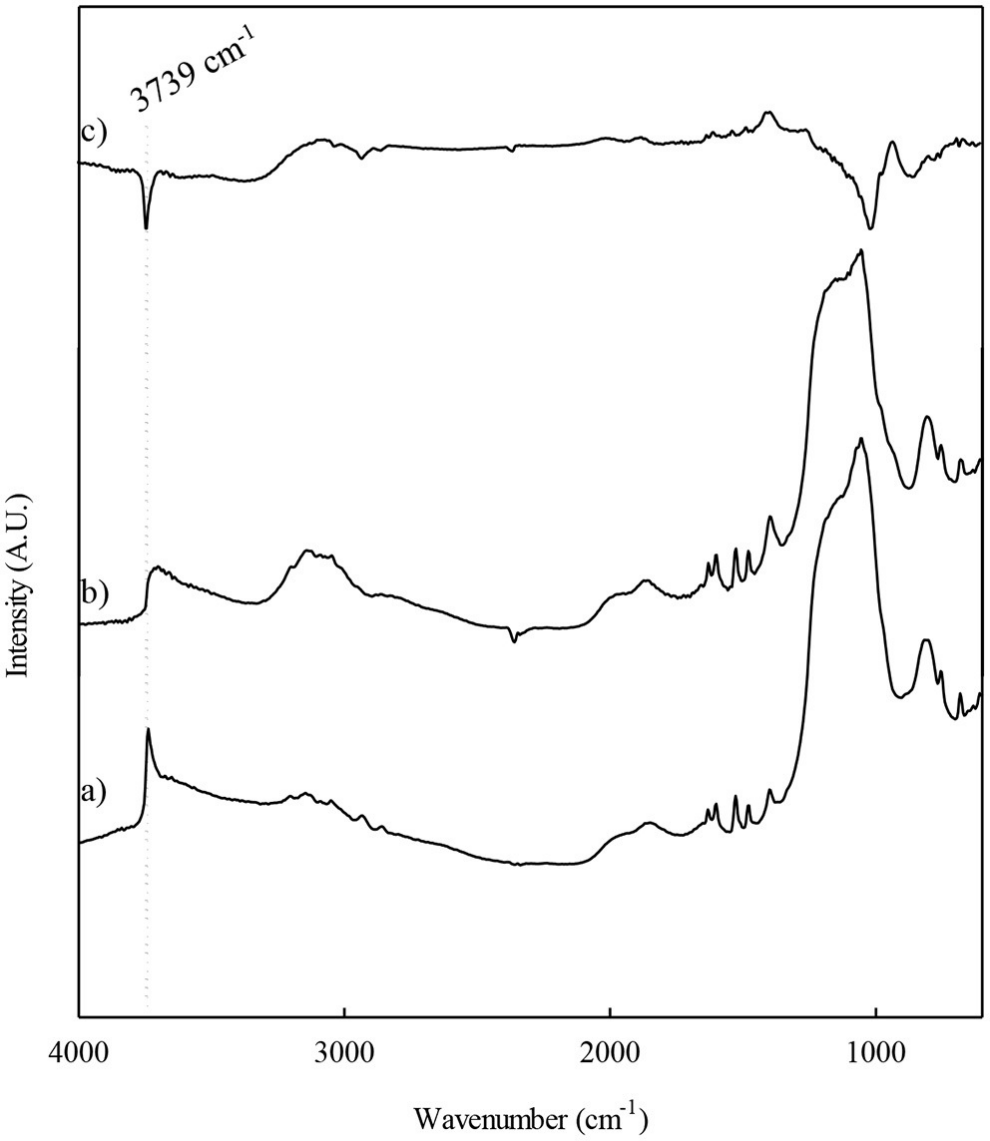
DRIFT spectra corresponding to the materials: (a) Al-SBA-15, (b) F10%-Nb1%/Al-SBA-15 and (c) differential spectrum.

The modifications on the catalysts surface after the different modifications that the Al-SBA-15 material used as support undergoes were investigated by DRIFT. On Figure 2, it can be observed a signal at 3,739 cm^−1^ corresponding the stretching of isolated -OH, typical for silanol groups. In addition, it is important to remark the presence of a wide band at 3,580 cm^−1^ that is related to possible to surface hydroxyl groups disturbed by hydrogen bonds with surface hydroxyl groups (Si-OH, Al-OH). The incorporation of either niobium or fluoride lead to a decrease in the bands related to hydroxyl groups. This decrease is more noticeable if the samples loaded with Nb and/or F are compared with their respective support, the Al-SBA-15 material. Therefore, the differential spectrum shows negative signals due to the growing of surface niobium or fluorine species. This fact points out that the incorporation of niobium as well as fluorine takes place over the silanol groups. In addition, it is clear the dihydroxylation, accompanied by the formation of water molecules that may hydrolyze the metal salt precursor (Pineda et al., 2011). Thus, it would be also possible the replacement of hydroxyl groups by fluoride anions. Accordingly, the incorporation of niobium as well as fluorine diminishes the amount of hydroxyl groups, but even at high F loadings (10 wt.%) a part of the hydroxyl groups remains in the material. Then, it could be predicted that such OH groups persisting on the material must be strong acid sites able to protonate 2,6-dimethylpyridine (Oliveiro et al., 2005).

The textural properties of the as-synthesized materials are shown on Table 1 and were evaluated through nitrogen adsorption/desorption measurements at liquid nitrogen temperature (77 K). The materials herein investigated loaded with niobium and/or fluorine as well as their respective support present isotherm type IV according to the IUPAC classification, usually found for mesoporous materials (Figure S2). Remarkably, the parent material, Al-SBA-15, used as support owns a high surface area, which declines after the incorporation of niobium and/or fluorine. As it could be expected such decrease in surface area is going to be more pronounced for those materials with the highest fluorine loading (10 wt.%). Thus, considering the results obtained by XRD it could be said that the treatment with high amounts of ammonium fluoride cause a deterioration in the materials structure, preserving the most characteristic textural and structural properties of SBA-15 materials. In addition, it can be observed that the materials with a highest fluorine loading present increased pore diameter and lower pore volume. Such behavior has been already reported by Luque et al., who observed a similar trend for Al-MCM-41 materials. The reason that could justify how textural properties are affected by the impregnation with ammonium fluoride was explained by Xu and coworkers that found out that the treatment with high loading of ammonium fluoride damage the mesoporous structure, leading to the formation of an excess of silanol groups (Xu et al., 2002). On the other side, the incorporation of Nb cause a decrease in surface area as compared with the Al-SBA-15 material used as support as well as a lower pore volume due to a partial closure of the pores by niobium oxide nanoparticle over the catalytic support pores.

**Table 1:** Textural properties evaluated using N_2_ adsorption/desorption measurements for representative materials prepared in this work.

**Materials**	**S_**BET**_ (m^**2**^g^**−1**^)**	**C_**BET**_**	**D_**BJH**_ (nm)**	**D^a^ (nm)**	**${\text{V}}_{\text{meso}}^{\text{b}}$ (cm^**3**^g^**−1**^)**	**V_**BJH**_ (cm^**3**^g^**−1**^)**
Al-SBA-15	812	87	7.9	8.1	0.84	1.85
F3%/Al-SBA-15	555	82	6.7	8.8	0.79	0.88
F10%/Al-SBA-15	291	53	10.5	10.1	0.77	0.89
Nb1%/Al-SBA-15	685	126	6.6	8.0	0.33	0.82
F3%-Nb1%/Al-SBA-15	382	62	6.9	8.8	0.53	0.64
F10%-Nb1%/Al-SBA-15	322	54	7.5	9.0	0.52	0.64

a *Pore volume determined in the adsorption branch*.

*^b^Mesopore volume*.

The acidic properties of the materials herein investigated were analyzed firstly by a chromatographic pulse method using pyridine and 2,6-dimethyl pyridine as probe molecules at 300°C, for the quantification of the number of acid sites. Secondly, the strength of such acid sites has been evaluated by temperature programmed desorption of pyridine (TPD-Py). Regarding to the evaluation of the number of acid sites two different bases have been employed, at first instance, pyridine that interacts with both Lewis and Brönsted acid sites and 2,6-dimethyl pyridine that it is coordinated more selectively with Brönsted acid sites. Therefore, Lewis acidity was determined easily by the difference between total and Brönsted acidity. The results obtained, displayed on Table 2, reveal a noticeable decrease in Brönsted acid sites after the incorporation either of niobium or fluorine as compared with the Al-SBA-15 material used as support. Regarding to Lewis acidity there are no appreciable differences among Nb and/or fluorine loaded materials. Therefore, these results will not allow, initially, to predict the catalytic activity in the valeric acid esterification reaction of the materials investigated in this work.

**Table 2:** Surface acid properties of the modified SBA-15 materials with niobium and/or fluorine evaluated using a pulse chromatographic titration method.

**Catalyst**	**Total acidity** **(μmoles piridine/g)**	**Brönsted acidity** **(μmoles dimethyl pyridine/g)**	**Lewis acidity** **(μmoles/g)**	**B/L**
Al-SBA-15	158	92	66	1.39
F3%/Al-SBA-15	129	55	74	0.74
F10%/Al-SBA-15	115	58	57	1.02
Nb1%/Al-SBA-15	133	74	59	1.25
F3%-Nb1%/Al-SBA-15	120	61	59	1.03
F10%-Nb1%/Al-SBA-15	95	43	52	0.83

**Figure 3:**
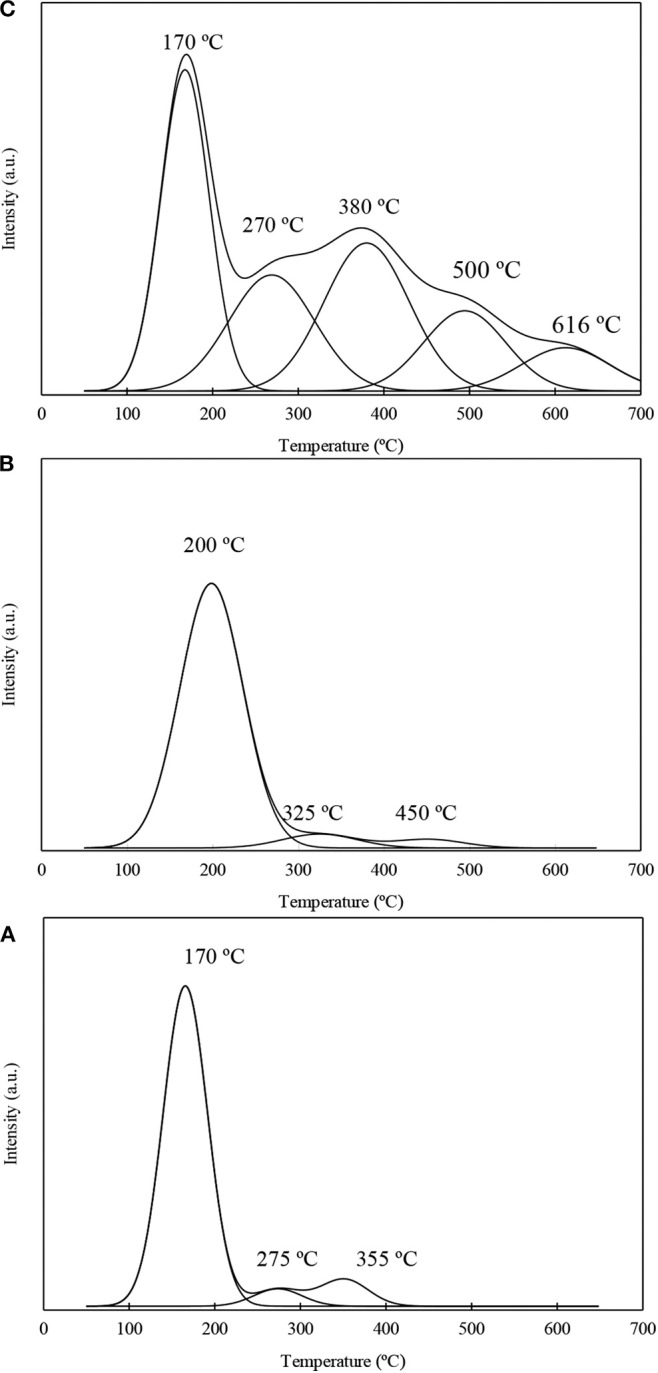
Py-TPD profiles corresponding to the samples: **(A)** Al-SBA-15, **(B)** Nb1%/Al-SBA-15, **(C)** F10%-Nb1%/Al-SBA-15.

Py-TPD experiments were conducted to evaluate the acidity strength of the acid sites on the niobium and/or fluorine loaded materials as well as for the material Al-SBA-15 used as support. Py-TPD profiles corresponding to the most representative samples investigated in this work are shown on Figure 3. The TPD profiles were deconvoluted in the different components assuming to be a Gaussian distribution. In all the cases, the materials investigated in this work present low temperature peak (<200°C) that corresponds to pyridine adsorbed on weak sites, which is the most important peak for the materials Al-SBA-15 and Nb1%/Al-SBA-15. In addition, medium strength acid sites, between 200 and 400°C, are residual for Al-SBA-15 and Nb1%/Al-SBA-15 samples while for the samples treated with ammonium fluoride these peaks appear with higher relative intensity. The most noticeable result obtained by Py-TPD is the confirmation of the formation of the strong acid sites on the fluorinated materials, merging two new signals above 500°C, not observable for the Al-SBA-15 and Nb1%/Al-SBA-15 materials. The distribution among weak, very weak, strong, and very strong has been calculated and displayed on Table S3.

The impact of the modification with Nb and/or F of the parent Al-SBA-15 on the acidity has been evaluated in the esterification of valeric acid with different alcohols, mainly ethanol, giving ethyl valerate as main product and water as the only side product. The reactions were assisted by microwave radiation, which provides a more direct and effective heating as compared to conventional reactors, using a power of 300 W for 15 min, reaching pressure values up to 4 bars. In addition, to thermodynamically favor the reaction an excess of the alcohol, used as solvent, has been employed (García-Sancho et al., 2017).

In agreement with results already published by other authors (Kresge et al., 1992; Aranda et al., 2009), the esterification of valeric acid with ethanol or methanol, two short chain alcohols, a better conversion was achieved when methanol (Table 3). However, ethanol was used for the following experiments due to its greener credentials as compared with methanol. Another important parameter investigated was the reaction temperature, which has been observed to have an exponential influence and was evaluated using the Nb0.5%/Al-SBA-15 catalyst. Thus, according to the results observed, 120°C has been choose as the optimum temperature, temperature below lead to low valeric acid conversion while a high temperature may cause an overpressure and make the process economically non-feasible.

**Table 3:** Influence of the use of different solvents and reaction temperatures in the valeric acid conversion when the reaction is catalyzed by niobium loaded catalysts.

**Catalyst**	**Temperature (°C)**	**Solvent**	**Conversion (%)**
Nb0.5%/AL-SBA-15	120	Methanol	40
Nb0.5%/AL-SBA-15	120	Ethanol	19
Nb0.5%/AL-SBA-15	80	Ethanol	<5
Nb0.5%/AL-SBA-15	100	Ethanol	<10
Nb0.5%/AL-SBA-15	150	Ethanol	35
Nb1%/Al-SBA-15	120	Ethanol	17

Reaction conditions: 50 mg de catalyst, 2 mL ethanol, 0.1 mL valeric acid, 120°C, microwave power: 300 W, irradiation time: 15 min.

On the contrary that it could be expected, of the incorporation of niobium on the mesoporous Al-SBA-15 materials, independently of the metal loading, affects negatively to the conversion of valeric acid in the esterification reaction with methanol, decreasing from a 30% to values around 17% for the niobium containing catalysts. Such undesired effect can be explained by the decrease of Brönsted acidity upon the incorporation of niobium that may hinder the access of the reactant molecules to the active sites in the valeric acid esterification existing on the aluminosilicate used as support. These results found for the Nb-based catalyst are contradictory with the results found in the literature (García-Sancho et al., 2017; Silva et al., 2017) for analogous materials but quite consistent with the characterization results.

**Figure 4:**
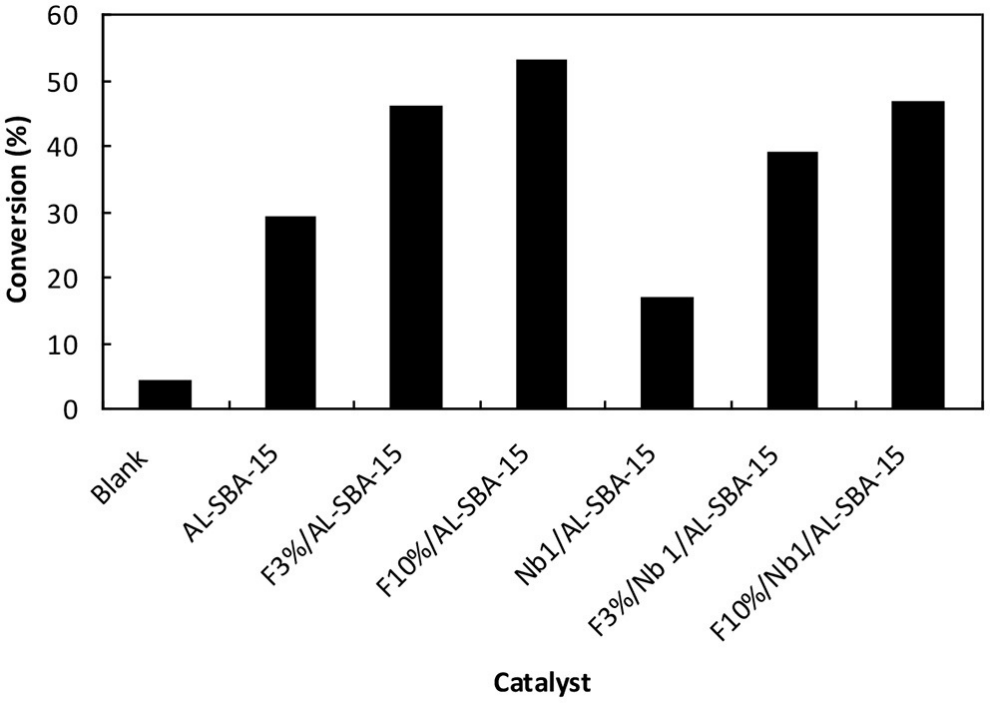
Catalytic activity of the materials, fluorinated and non-fluorinated materials investigated in this work in the esterification of valeric acid with ethanol. Reaction conditions: 50 mg de catalyst, 2 mL ethanol, 0.1 mL valeric acid, 120°C, microwave power: 300 W, irradiation time: 15 min.

On the other hand, the incorporation of fluorine on Al-SBA-15 as well as on Nb1/Al-SBA-15 cause a remarkable on the catalytic performance. As it can be observed on Figure 4 (see also Table S2), those materials modified fluorine exhibited a superior catalytic activity in the microwave-assisted esterification of valeric acid with methanol. Such increase in the catalytic activity is proportional to the amount of fluorine loaded on the material in both cases for Al-SBA-15 and Nb1/Al-SBA-15 materials. Thus, in the case of F10%/Al-SBA-15 the conversion increased up to the 55% almost twice as compare with the conversion obtained by the use of non-fluorinated Al-SBA-15. Notwithstanding, the catalytic activity of the material Nb1%/Al-SBA-15 loaded with fluorine is still lower than the achieved through the use of analogous material without niobium.

Remarkably, the most relevant catalysts herein investigated, F10%/Al-SBA-15 and F10%-Nb1%/Al-SBA-15, revealed a high stability in the reaction conditions evaluated without observing any decrease in the conversion achieved by these materials in the valeric acid esterification after 4 reuses (Figure S4).

According to the literature the results herein reported are quite far of being the best in terms of catalytic activity. However, the fact that lipases have been found mostly to catalyze the esterification of pentanoic acid make difficult to make a fair comparison. Thus, our research group has reported almost quantitative conversion of valeric acid by biosilicified enzymes at 40°C after in a higher reaction time as compared with the time employed here for the catalyst screening. However, such has encountered certain limitations such as the temperature and that for example the use of methanol may poison the enzyme (Cebrián-García et al., 2018b). Therefore, the fluorinated catalysts herein synthesized provide an alternative to enzymes for the production of valerate esters more robust and with less limitations as compared with lipases.

### Conclusions

The synthesis and characterization of SBA-15 type catalysts loaded with niobium and/or fluorine has been carried out in this work. With regard to niobium containing systems did not experience any significant changes in terms of textural/structural properties, although a slight deterioration was evidenced as compared with the parent material. The subsequent modification with fluorine did not cause any effect in structural properties at low concentrations of fluorine, although significant changes (deterioration of the hexagonal arrangement of the structure and pore diameter increase, mainly) are evidenced at large F contents (10 wt.%). Such modifications had a higher impact on surface acid properties, modifications with both Nb and F lead to a decrease in Brönsted acid sites as well as a loss in hydroxyl groups. The most important effect was found after the incorporation of fluorine when hydroxyl groups were partially substituted by F, in principle giving rise to stronger acid sites.

The catalytic performance of the as-synthesized materials was investigated in the esterification of valeric or pentanoic acid. While the materials loaded with niobium displayed lower catalytic activity as compared with the support, Al-SBA-15, that has been attributed to the blockage of the active sites by niobium particles.

In view of its further utilization in the esterification reaction of valeric acid and ethanol, giving as a product ethyl valerate. F10%/Al-SBA-15 exhibited optimum product yields under the investigated reaction conditions. In contrast, the catalyst modified with niobium or niobium and fluorine presented a catalytic activity lower as compared to the catalyst modified only with fluorine.

Thus, it an be concluded, firstly, that the main factor to improve the catalytic activity in the valeric acid esterification reaction is clearly the presence of stronger Brönsted acid sites and, secondly, that the addition of Nb rendered catalysts with decreased catalytic activities, contrary to expectations in the target esterification.

## Data Availability Statement

All datasets generated for this study are included in the article/Supplementary Material.

## Author Contributions

MB-S and NL conducted all experiments in the lab. AP and EP wrote the first draft of the manuscript. AB and RL planned and programmed all experiments and wrote the final manuscript including the discussion.

### Conflict of Interest

The authors declare that the research was conducted in the absence of any commercial or financial relationships that could be construed as a potential conflict of interest.

## References

[B1] Aranda D. A. G. de Araújo Gonçalves J. Peres J. S. Dantas Ramos A. L. Ribeiro de Melo C. A. Jr. Antunes Nelson O. A. C. (2009). The use of acids, niobium oxide, and zeolite catalysts for esterification reactions J. Phys. Org. Chem. 22, 709–716. 10.1002/poc.1520

[B2] Borges M. E. Díaz L. (2012). Recent developments on heterogeneous catalysts for biodiesel production by oil esterification and transesterification reactions: a review. Renew. Sustain. Energy Rev. 16, 2839–2849. 10.1016/j.rser.2012.01.071

[B3] Carmo A. C. Jr. de Souza L. K. da Costa C. E. Longo E. Zamian J. R. da Rocha Filho G. N. (2008). Production of biodiesel by esterification of palmitic acid over mesoporous aluminosilicate Al-MCM-41. Fuel 88, 461–468. 10.1016/j.fuel.2008.10.007

[B4] Cebrián-García S. Balu A. García A. Luque R. (2018b). Sol-gel immobilisation of lipases: towards active and stable biocatalysts for the esterification of valeric acid. Molecules 23:2283. 10.3390/molecules23092283 30200657 PMC6225346

[B5] Cebrián-García S. Balu A. M. Luque R. (2018a). Ultrasound-assisted esterification of valeric acid to alkyl valerates promoted by biosilicified lipases. Front. Chem. 6:197. 10.3389/fchem.2018.00197 29930937 PMC5999784

[B6] Corradini M. C. C. Costa B. M. Bressani A. P. P. Garcia K. C. A. Pereira E. B. Mendes A. A. (2016). Improvement of the enzymatic synthesis of ethyl valerate by esterification reaction in a solvent system. Prep. Biochem. Biotech. 47, 100–109. 10.1080/10826068.2016.1181084 27136358

[B7] Doyle A. M. Albayati T. M. Abbas A. S. Alismaeel Z. T. (2016). Biodiesel production by esterification of oleic acid over zeolite Y prepared from kaolin. Renew. Energy 97, 19–23. 10.1016/j.renene.2016.05.067

[B8] García-Sancho C. Saboya R. Cecilia J. Sales A. Luna F. Rodríguez-Castellón E. (2017). Influence of pore size and loading for Nb_2_O_5_/SBA-15 catalysts on synthetic ester production from free fatty acids of castor oil. Mol. Catal. 436, 267–275. 10.1016/j.mcat.2017.04.036

[B9] Ho D. P. Ngo H. H. Guo W. (2014). A mini review on renewable sources for biofuel. Bioresour. Technol. 169, 742–749. 10.1016/j.biortech.2014.07.022 25115598

[B10] Jarry B. Launay F. Nogier J. P. Montouillout V. Gengembre L. Bonardet J. L. (2006). Catalytic activity of mesoporous Ga-SBA-15 materials in α-pinene isomerisation: similarities and differences with Al-SBA-15 analogues. Appl. Catal. A 309, 177–186.

[B11] Jehng J. M. Wachs I. E. (1991). The molecular structures of supported niobium oxide catalysts under in situ conditions. J. Phys. Chem. 95, 7373–7379. 10.1002/chin.199151025

[B12] Kon K. Onodera W. Shimizu K. I. (2014). Selective hydrogenation of levulinic acid to valeric acid and valeric biofuels by a Pt/HMFI catalyst. Catal. Sci. Technol. 4, 3227–3234. 10.1039/C4CY00504J

[B13] Kresge C. T. Leonowicz M. E. Roth W. J. Vartuli J. C. Beck J. S. (1992). Ordered mesoporous molecular sieves synthesized by a liquid-crystal template mechanism. Nature 359, 710–712. 10.1038/359710a0

[B14] Lange J. P. Price R. Ayoub P. M. Louis J. Petrus L. Clarke L.. (2010). Valeric biofuels: a platform of cellulosic transportation fuels. Angew. Chem. Int. Ed. 49, 4479–4483. 10.1002/anie.201000655 20446282

[B15] López D. E. Goodwin J. G. Jr. Bruce D. A. Furuta S. (2008). Esterification and transesterification using modified zirconia catalysts. Appl. Catal. A 339, 76–83. 10.1016/j.apcata.2008.01.009

[B16] Luque R. Balu A. M. Campelo J. M. Gracia M. D. Losada E. Pineda A. (2012). Catalytic applications of mesoporous silica-based materials. Catalysis 24, 253–280 10.1039/9781849734776-00253

[B17] Luque R. Campelo J. M. Luna D. Marinas J. M. Romero A. A. (2005). NH_4_F effect in post-synthesis treatment of Al-MCM-41 mesoporous materials. Micropor. Mesopor. Mater. 84, 11–20. 10.1016/j.micromeso.2005.05.013

[B18] Mo X. Lotero E. J. Changqing L. Yijun L. Goodwin J. G. Jr. (2008). A novel sulfonated carbon composite solid acid catalyst for biodiesel synthesis. Catal. Let. 123, 1–6. 10.1007/s10562-008-9456-y

[B19] Naika S. N. Goud V. V. Rout P. K. Dalai A. K. (2010). Production of first and second generation biofuels: a comprehensive review. Renew. Sustain. Energy Rev. 14, 578–597, 10.1016/j.rser.2009.10.003

[B20] Oliveiro L. Vimont A. Lavalley J. C. Romero-Sarria F. Gaillard M. Maugé F. (2005). 2,6-Dimethylpyridine as a probe of the strength of Brønsted acid sites: study on zeolites. Application to alumina. Phys. Chem. Chem. Phys. 7, 1861–1869. 10.1039/B500689A 19787950

[B21] Palkovits R. (2010). Pentenoic acid pathways for cellulosic biofuels. Angew. Chem. Int. Ed. 49, 4336–4338. 10.1002/anie.201002061 20480484

[B22] Park J. Kim D. Lee J. (2010). Esterification of free fatty acids using water-tolerable Amberlyst as a heterogeneous catalyst. Bioresour. Technol. 101, 62–65. 10.1016/j.biortech.2009.03.035 19362818

[B23] Pineda A. Balu A. M. Campelo J. M. Romero A. A. Carmona D. Balas F.. (2011). A dry milling approach for the synthesis of highly active nanoparticles supported on porous materials. ChemSusChem 4, 1561–1565. 10.1002/cssc.201100265 22191094

[B24] Pineda A. Ojeda M. Romero A. A. Balu A. M. Luque R. (2018). Mechanochemical synthesis of supported cobalt oxide nanoparticles on mesoporous materials as versatile bifunctional catalysts. Micropor. Mesopor. Mater. 272, 129–136, 10.1016/j.micromeso.2018.06.029

[B25] Rade L. L. Lemos C. O. T. Barrozo M. A. S. Ribas R. M. Monteiro R. S. Hori C. E. (2018). Optimization of continuous esterification of oleic acid with ethanol over niobic acid. Renew. Energy 115, 208–216. 10.1016/j.renene.2017.08.035

[B26] Sharma M. Toor A. P. Wanchoo R. K. (2014). Kinetics of the esterification reaction between pentanoic acid and methanol catalyzed by noncorrosive cation exchange resin. Chem. Biochem. Eng. Q. 28, 79–85. 10.15255/CABEQ.2014.32

[B27] Sheldon R. A. (2016). Green chemistry, catalysis and valorization of waste biomass. J. Mol. Catal. A-Chem. 422, 3–12. 10.1016/j.molcata.2016.01.013

[B28] Silva Â. Wilson K. Lee A. F. Santos V. C. D. Bacilla A. C. C. Mantovani K. M. (2017). Nb2O5/SBA-15 catalyzed propanoic acid esterification. Appl. Catal. B Environ. 205, 498–504. 10.1016/j.apcatb.2016.12.066

[B29] Srilatha K. Lingaiah N. Prabhavathi Devi B. L. A. Prasad R. B. N. Venkateswar S. Sai Prasad P. S. (2009). Esterification of free fatty acids for biodiesel production over heteropoly tungstate supported on niobia catalysts. Appl. Catal. A 265, 28–33. 10.1016/j.apcata.2009.05.025

[B30] Stuckv G. Zhao D. Yang P. Lukens W. Melosh N. Chmelka B. (1998). Using the organic-inorganic interface to define pore and macroscale structure. Stud. Surf. Sci. Catal. 117, 1–12. 10.1016/S0167-2991(98)80972-5

[B31] Sun P. Gao G. Zhao Z. Xia C. Li F. (2014). Stabilization of cobalt catalysts by embedment for efficient production of valeric biofuel. ACS Catal. 4, 4136–4142. 10.1021/cs501409s

[B32] Udayakumar V. Pandurangan A. (2014). Catalytic activity of mesoporous V/SBA-15 in the transesterification and esterification of fatty acids. J. Porous Mater. 21, 921–931. 10.1007/s10934-014-9839-y

[B33] Xu M. Wang W. Seiler M. Buchholz A. Hunger M. (2002). Improved brønsted acidity of mesoporous Al-MCM-41 material treated with ammonium fluoride^†^. J. Phys. Chem. B. 106, 3202–3208. 10.1021/jp014222a

[B34] Zhao D. (1998). Triblock copolymer syntheses of mesoporous silica with periodic 50 to 300 angstrom pores. Science 279, 548–552. 10.1126/science.279.5350.548 9438845

[B35] Zhao D. Huo Q. Feng J. Chmelka B. F. Stucky G. D. (2014). Nonionic triblock and star diblock copolymer and oligomeric surfactant syntheses of highly ordered, hydrothermally stable, mesoporous silica structures. J. Am. Chem. Soc. 136, 10546–10546. 10.1021/ja506344k

