# Supplementary material for: Tuneable Acidity in Fluorinated Al-SBA-15 Materials for the Esterification of Valeric Acid to Alkyl Valerates
Source: Front Chem. 2020 Jan 31;8:42. doi: 10.3389/fchem.2020.00042 (PMC7005226; doi:10.3389/fchem.2020.00042)

Supplementary Material

Tuneable acidity in fluorinated Al-SBA-15 Materials for the Esterification of Valeric Acid to Alkyl Valerates

Miguel Blanco-Sánchez^1^, Evan Pfab^1^, Noelia Lázaro^1^, Antonio Pineda^1^, Alina M. Balu^1^, and Rafael Luque^1,2*^

^1^Departamento de Química Orgánica, Universidad de Córdoba, Edif. Marie Curie, Ctra. Nnal. IV-A, Km 396, E14014 Córdoba, Spain;

^2^ Chemistry Department, Peoples Friendship University of Russia (RUDN University), 6 Miklukho-Maklaya Str., 117198 Moscow, Russia

*** Correspondence:**Rafael Luque
q62alsor@uco.es

Keywords: heterogeneous catalysts; mesoporous materials; biomass valorisation; esterification;

alkyl valerates

**Figure S1.** Low-angle X-ray diffractograms for the material Nb1%/Al-SBA-15 (a), and for the materials obtained with different fluorine loadings: F3%-Nb1%/Al-SBA-15 (b) and F10%-NB1%/Al‑SBA‑15 (c).


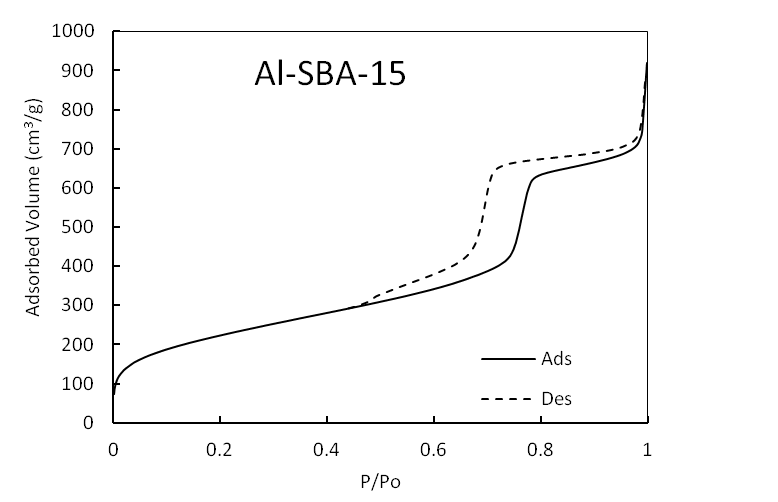

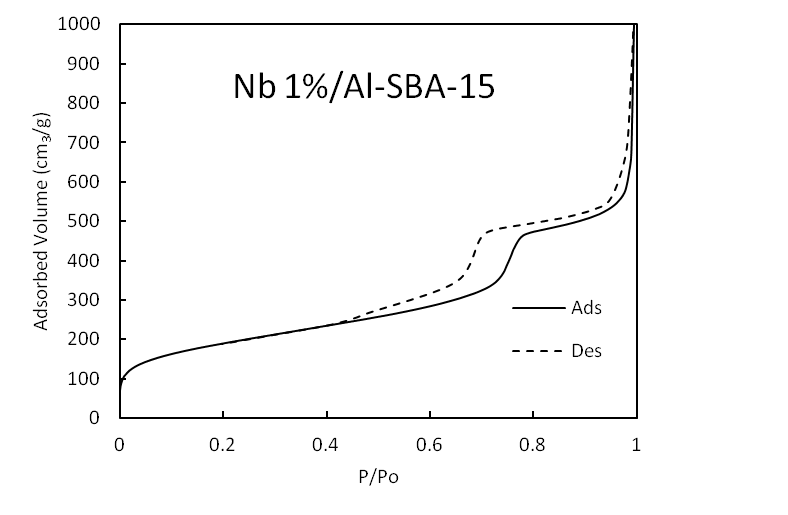

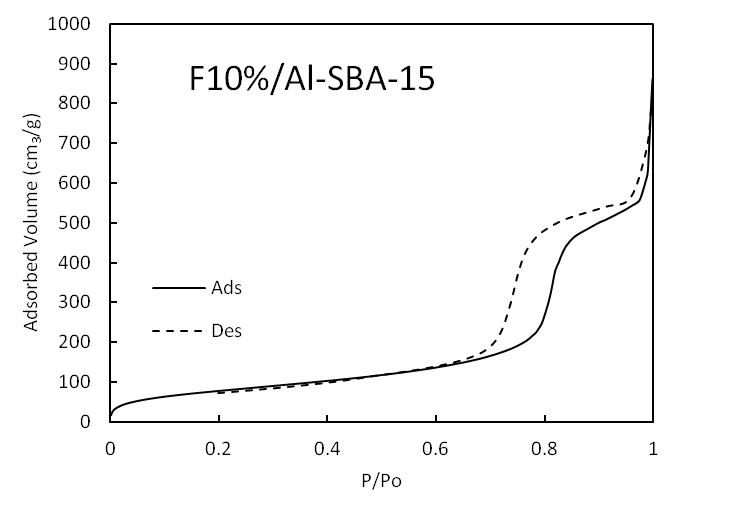

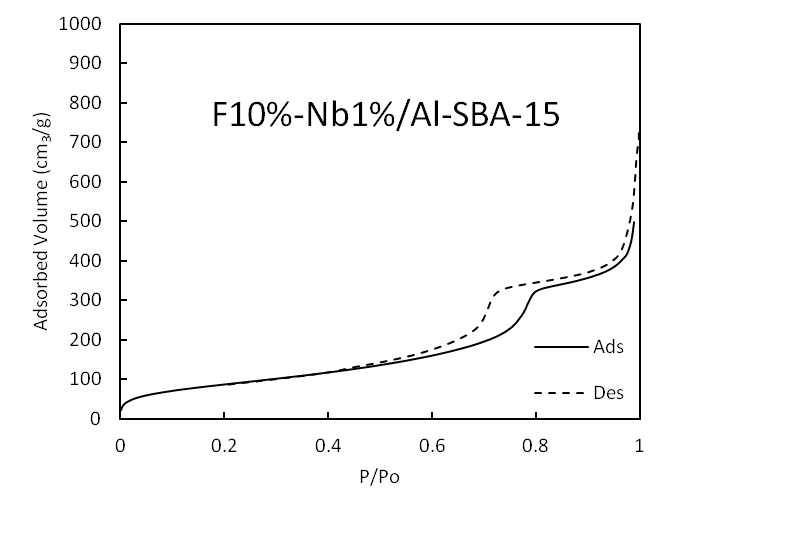


**Figure S2.** Nitrogen adsorption-desorption plots corresponding to the materials Al-SBA-15, Nb1%/Al-SBA-15, F10%/Al-SBA-15 y F10%-Nb1%/Al-SBA-15

**Table S1:** Elemental analysis for the selected samples obtained by ICP-MS.

| **Materials** | | | | **Si/Al**  **atomic**  **ratio** | | | **Elemental análisis (wt.%)** | | | | | | |
| --- | --- | --- | --- | --- | --- | --- | --- | --- | --- | --- | --- | --- | --- |
|  |  |  |  |  |  |  | **Al** | | **Nb** | | | | **F** |
| Al-SBA-15 | | | | 19.2 | | | 1.1 | - | | | | - | |
| F3%/Al-SBA-15 | | | | 16.7 | | | 1.2 | - | | | | 2.2 | |
| F10%/Al-SBA-15 | | | | 12.6 | | | 1.3 | - | | | | 8.1 | |
| Nb1%/Al-SBA-15 | | | | 19.1 | | | 1.1 | 0.66 | | | | 8.0 | |
| F3%‑Nb1%/Al‑SBA‑15 | | | | 17.0 | | | 1.2 | 0.64 | | | | 2.5 | |
| F10%‑Nb1%/Al‑SBA‑15 | | | | 14.1 | | | 1.2 | 0.61 | | | | 7.0 | |
|  | **Table S2:** Catalytic activity (TOF) in the valeric acid esterification obtained by the materials investigated in this work as well as the standard deviation (SD) in the experiments performed to get the data displayed on Figure 4. | | | | | | | | | |  | | |
|  | **Material** | | **TOF**  **(mol converted /mol H^+^ h)** | | | **SD**  **(%)** | | | | |  | | |
|  | Al-SBA-15 | 66 | | | 2.2 | | | | |  | | | |
|  | F3%/Al-SBA-15 | 128 | | | 1 | | | | |  | | | |
|  | F10%/Al-SBA-15 | 166 | | | 2 | | | | |  | | | |
|  | Nb1%/Al-SBA-15 | 46 | | | 4 | | | | |  | | | |
|  | F3%-Nb1%/Al-SBA-15 | 117 | | | 3 | | | | |  | | | |
|  | F10%-Nb1%/Al-SBA-15 | 177 | | | 3 | | | | |  | | | |

**Table S3.** Relative areas obtained by Py-TPD for the different acid sites existing in the investigated materials.

| **Catalyst** | **Acidity Strength** | | | | **TOF**  **(mol converted /mol H^+^ h)** |
| --- | --- | --- | --- | --- | --- |
|  | **Very weak**  **(%)** | **Weak**  **(%)** | **Strong**  **(%)** | **Very Strong**  **(%)** |  |
| Al-SBA-15 | 84.3 | 3.8 | 7.6 | 4.3 | 66 |
| Nb1/Al-SBA-15 | 91.2 | 5.6 | 3.2 | 0.0 | 46 |
| F10-Nb1/Al-SBA-15 | 39.2 | 16.7 | 28.9 | 15.2 | 166 |

**Figure S3.** Wide-angle XRD corresponding to the material Nb1%/Al-SBA-15.


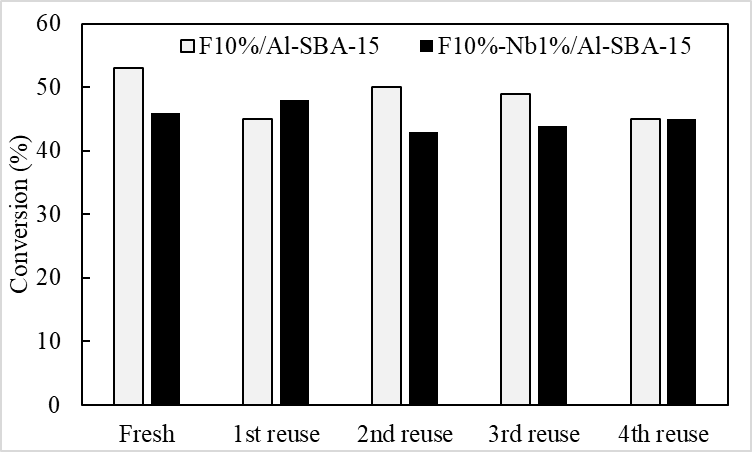


Figure S4. Valeric acid conversion achieved by F10%/Al-SBA-15 and F10%-Nb1%/Al-SBA-15 in the esterification reaction with ethanol after several catalytic cycles. Reaction conditions: Reaction conditions: 50 mg de catalyst, 2 mL ethanol, 0.1 mL valeric acid, 120 °C, microwave power: 300 W, irradiation time: 15 minutes.


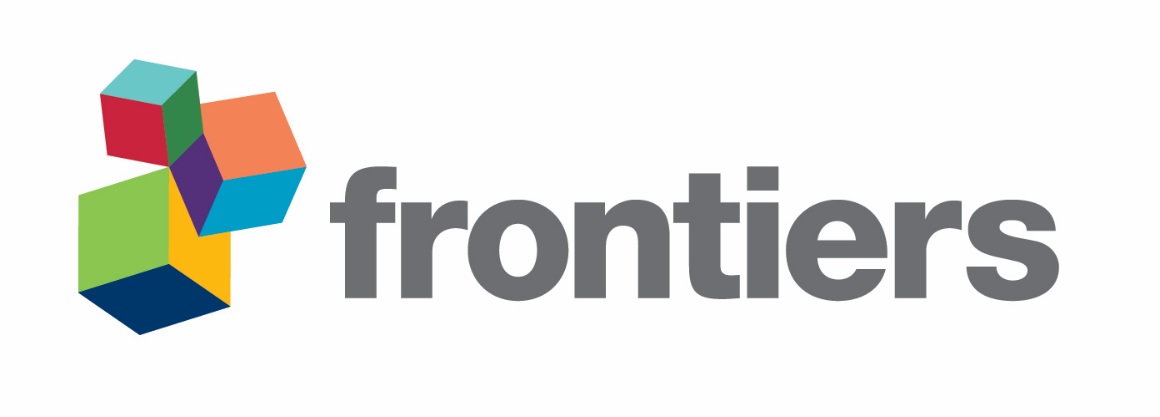

Supplement: Supplementary file 1 [file Data_Sheet_1.doc]
